# FoxD3-regulated microRNA-137 suppresses tumour growth and metastasis in human hepatocellular carcinoma by targeting AKT2

**DOI:** 10.18632/oncotarget.2089

**Published:** 2014-06-10

**Authors:** Li-Li Liu, Shi-Xun Lu, Min Li, Lin-Zi Li, Jia Fu, Wen Hu, Yuan-Zhong Yang, Rong-Zhen Luo, Chris Zhiyi Zhang, Jing-Ping Yun

**Affiliations:** ^1^ Sun Yat-sen University Cancer Center; State Key Laboratory of Oncology in South China; Collaborative Innovation Center for Cancer Medicine; ^2^ Department of Pathology, Sun Yat-sen University Cancer Center

**Keywords:** miR-137, AKT2, FoxD3, metastasis, HCC

## Abstract

microRNAs, frequently deregulated in human cancer, have been implicated in the progression of hepatocarcinogenesis. Here, we show that microRNA (miR)-137 is significantly down-regulated in hepatocellular carcinoma (HCC). Its decreased expression is associated with vein invasion, incomplete involucrum, and distant metastasis. Multivariate analysis suggests that miR-137 is an independent indicator for poor survival. We next show that over-expression of miR-137 suppresses cell proliferation, migration and invasion *in vitro*. Conversely, miR-137 inhibition promotes HCC cell growth. We also identify AKT2 as a key target of miR-137 in this context. Statistical data reveal a reverse correlation of AKT2 and miR-137 expression in HCC patients. Silencing of AKT2 phenotypically copied miR-137-induced phenotypes, whereas re-expression of AKT2 reversed the suppressive effects of miR-137. Further investigations showed that miR-137 exerted its anti-tumour activity via inhibiting the AKT2/mTOR pathway. Moreover, we demonstrate that FoxD3 directly binds to the promoter of miR-137 and activates its transcription. *In vivo* studies confirm that FoxD3-regulated miR-137 inhibited HCC growth and metastasis via targeting AKT2. Together, our findings indicate that miR-137 is a valuable biomarker for HCC prognosis and the FoxD3/miR-137/AKT2 regulatory network plays an important role in HCC progression.

## INTRODUCTION

Hepatocellular carcinoma (HCC) is the third leading cause for cancer-related death worldwide [1]. Despite advances in HCC treatment, the 5-year overall survival rate remains very poor, due to the intrahepatic metastases or postsurgical recurrence [2]. The pathogenesis of HCC is not well defined, although preneoplastic liver lesions, hepatitis virus infection (HBV and HCV), alcohol abuse and aflatoxin exposure are associated with the carcinogenic process [3]. Thus, there is an urgent need to elucidate the mechanism of hepatocarcinogenesis to provide useful information for the clinical management of HCC.

microRNAs are ~22 nucleotides noncoding RNAs that negatively regulate gene expressions by degrading the target mRNA or repressing its protein translation [4]. Accumulating studies have suggested that deregulation of miRNAs have been linked to the development of a wide range of human diseases, including cancers. microRNA functions as either oncogene or tumour suppressor in different types of cancer [5]. In HCC, studies have also focused on the effect of miRNAs on tumour growth and metastasis. For example, miR-26a inhibits [6], whereas miR-17-5p promotes tumour growth and metastasis in HCC [7]. miR-137 functions primarily as an anti-tumour miRNA and is dysregulated in multiple types of cancer, including head and neck cancer [8] and colorectal cancer [9]. Aberrant epigenetic regulation of miR-137 promoter constitutes a key mechanism for miR-137 down-regulation [8]. Liang et al. demonstrated that transcriptional factor HMGA1 influenced miR-137 expression in colorectal cancer [9]. To date, the mechanism of miR-137 deregulation and its regulatory networks in HCC remain elusive.

AKT2 (v-AKT murine thymoma viral oncogene homologue 2), a pro-survival protein, is activated by the phosphatidylinositol 3' kinase (PI3K) pathway. The activation of the PI3K/AKT pathway is associated with aggressive phenotypes and poor outcomes in human cancers [10]. Mounting evidences show that AKT2 is involved in cancer development. Overexpression of AKT2 was frequently observed in HCC and breast cancer [11, 12]. Moro et al. reported that AKT2 was activated in prostate cancer cells in response to oxidative stress, resulting in enhanced cell migration and survival [13]. AKT2 has also been shown as an independent prognostic marker for the development and progression of HCC [11]. Recent studies indicate that AKT2 could be regulated by microRNAs. miR-708 targeted AKT2 to inhibit growth of prostate cancer [14]. miR-203 down-regulated AKT2 to sensitise p53-mutated colon cancer cells to chemotherapy [15].

In this study, we found that miR-137 expression was significantly down-regulated and associated with tumour metastasis and poor prognosis in HCC. We also demonstrated that the FoxD3/miR-137/AKT2 regulatory network played an important role in HCC progression.

## RESULTS

### Down-regulation of miR-137 is associated with metastasis and poor prognosis in HCC

**Figure 1:**
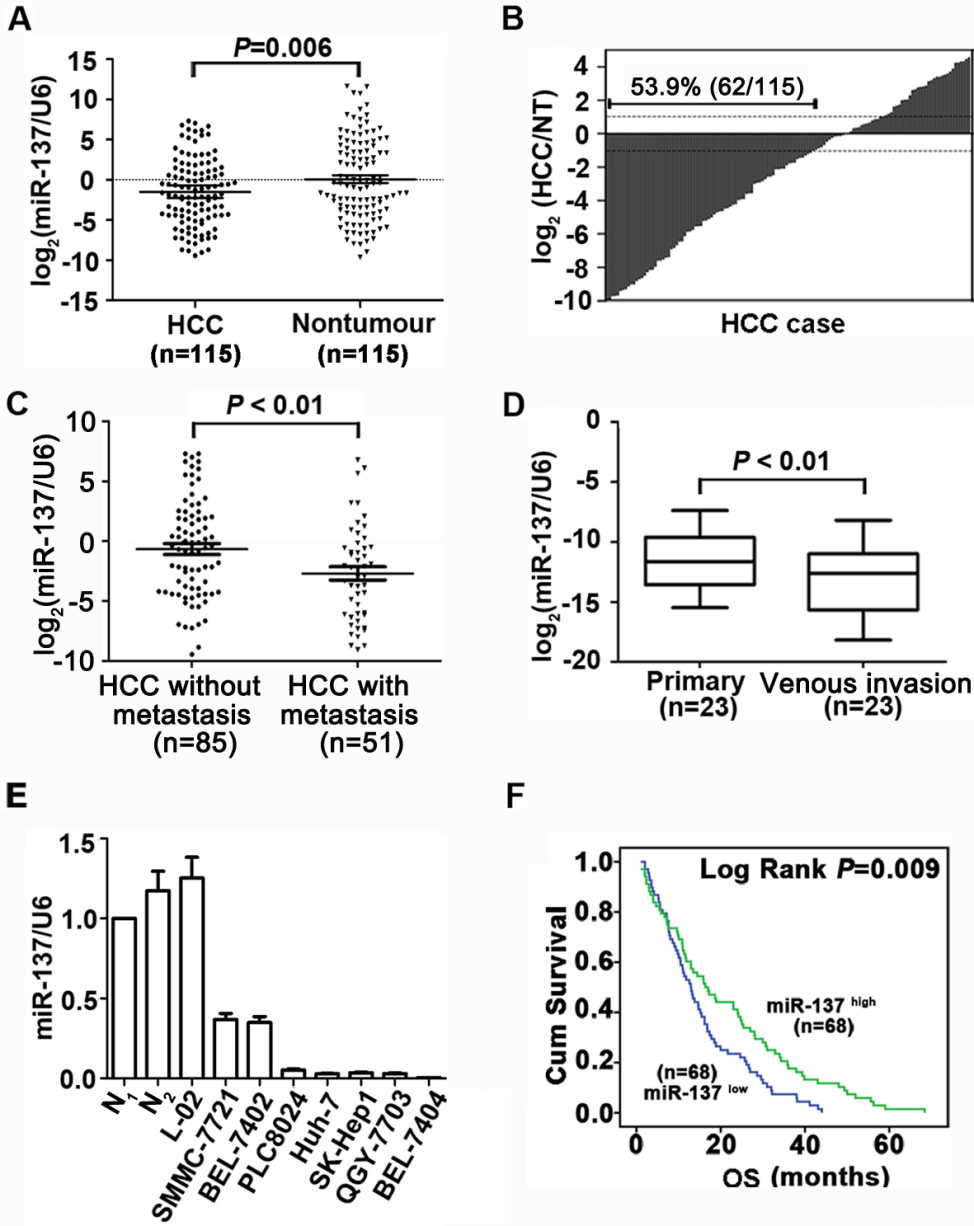
miR-137 is frequently down-regulated in HCC and associates with poor outcome (A, B) miR-137 expression was significantly decreased in HCC compared to the corresponding non-tumourous (NT) livers using qRT-PCR analyses. Expression was shown as a log_2_-fold change. (C) Scatter plots of the relative expression of miR-137 in patients without metastasis or with metastasis. 136 cases (venous invasion was observed in 29 patients and distant metastasis was observed in 22 patients) were subjected to qRT-PCR. Data were the mean ± SEM. (D) miR-137 expression was down-regulated in metastatic tumours (venous metastases) compared to primary HCC. Twenty-three cases were subjected to qRT-PCR. (E) miR-137 expression in 2 normal liver tissues, the immortalized liver cell line, L-02, and HCC cell lines. miR-137 expression was lower in HCC cell lines compared to normal liver (N_1_). Data were the mean ± SEM. (F) Decrease in miR-137 was significantly associated with the overall survival of 136 HCC patients. The median was used as the cut-off value to divide patients into low and high expression groups. All *P<0.05, **P<0.01.

Data of our miRNA array indicated the expression of miR-137 was down-regulated by 2.38-fold in HCC [16]. Using 115 pairs of primary HCC, we found that the median of miR-137 expression was 3-fold lower, compared to the non-tumourous tissues (median is −1.8386 and −0.4844, respectively) (Fig. 1A). Decreased expression of miR-137 in HCC was observed in 53.9% (62/115) of the cases (Fig. 1B). HCC patients who developed metastasis were with lower expression level of miR-137 (Fig. 1C). miR-137 expression was markedly reduced in HCC venous invasion (Fig. 1D). Furthermore, miR-137 expression in HCC cell lines was significantly decreased compared with the normal liver tissues (Fig. 1E). Clinical patients were divided into two groups according to the median of miR-137 expression in HCC samples. The down-regulation of miR-137 was significantly correlated with vein invasion, incomplete Involucrum and distant metastasis (Supplementary Table 1). Kaplan-Meier plots revealed that down-regulation of miR-137 was associated with unfavourable overall survival (Fig. 1F). Multivariate Cox regression analysis confirmed that miR-137 was an independent indicator for poor outcome (Supplementary Table 2). Taken together, these results suggest miR-137 as a potential biomarker for prognostic predictor in HCC.

### miR-137 inhibits proliferation and migration of HCC cells *in vitro*

**Figure 2:**
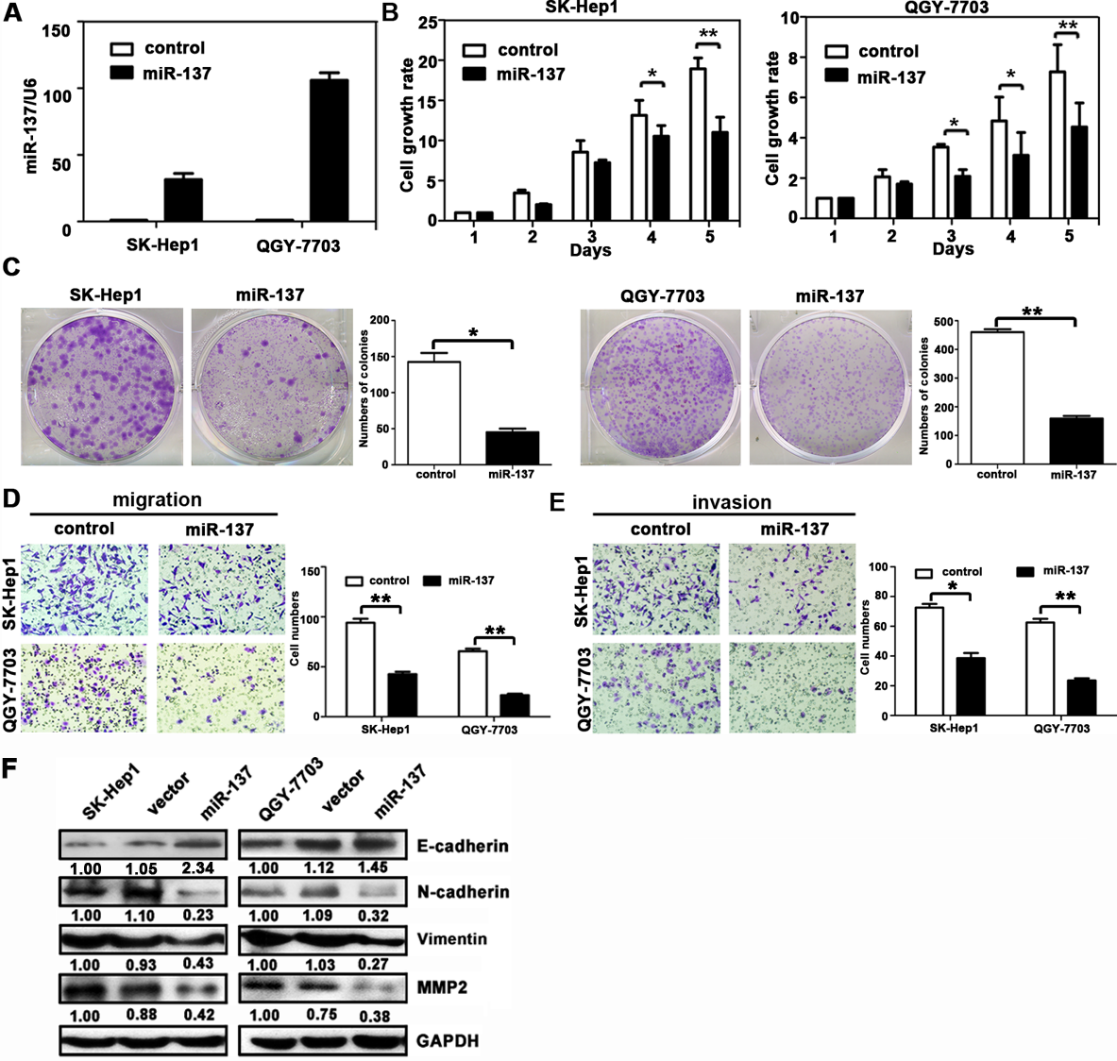
miR-137 inhibits HCC cell proliferation, migration and invasion *in vitro* (A) miR-137 re-expression was confirmed in SK-Hep1 and QGY-7703 cells using qRT-PCR. (B) miR-137 reduced cell viabilities in HCC. Cells were seeded onto 96-well plates and transfected with miR-137 mimics for 5 days. Cell viabilities were determined using MTT assays. (C) miR-137 suppressed colony formation in HCC. Cells stably expressing miR-137 were cultured with 400 μg/ml G418 for two weeks. The number of colonies was calculated and depicted by the histogramme. Data were represented as the mean + SEM of three independent experiments. (D, E) miR-137 inhibited cell migration and invasion in HCC. Transwell and invasion assays were performed to determine the effect of miR-137 on HCC cell migration and invasion. Representative images (left panel) and the quantification of three randomly selected fields (right panel) are shown. (F) The protein expression levels of E-cadherin, N-cadherin, vimentin, and MMP2 in SK-Hep1 and QGY-7703 cells transfected with miR-137 mimics for 24 h were examined. All *P < 0.05; ** *P* < 0.01.

To determine the biological function of miR-137 in HCC progression, we re-introduced miR-137 into SK-Hep1 and QGY-7703 cells. The expressions of miR-137 were determined (Fig. 2A). The cell viabilities of HCC cells infected with miR-137 mimics were significantly inhibited (Fig. 2B). Consistently, ectopic miR-137 expression dramatically suppressed colony formation (Fig. 2C). In contrast, miR-137 inhibition in SMMC-7721 and Bel-7402 cells remarkably increased cell proliferation and colony formation (Supplementary Fig. S1A-C). Our results suggested that miR-137 represses proliferation of HCC cells.

Given that miR-137 expression was associated with tumour metastasis in HCC patients, we next examined whether miR-137 could affect HCC cell migration and invasion. Results showed that miR-137 re-expression distinctly abrogated the migration and invasion of SK-Hep1 and QGY-7703 cells (Fig. 2D-E). Silencing of miR-137 in SMMC-7721 and Bel-7402 cells promoted the migration and invasion (Supplementary Fig. S1D-E). Western blot analysis showed that miR-137 over-expression resulted in decrease of p-AKT, p-mTOR, p-p70S6K, MMP2, and vimentin, but increase of E-cadherin (Fig. 2F and Supplementary Fig.S2B). In clinical samples, E-cadherin expression was lower, whereas vimentin level was higher in HCC patients with low miR-137 expression (Supplementary Fig.S2A).Our data indicated that miR-137 suppressed cell migration and invasion of HCC cells.

### AKT2 is a direct target of miR-137

**Figure 3:**
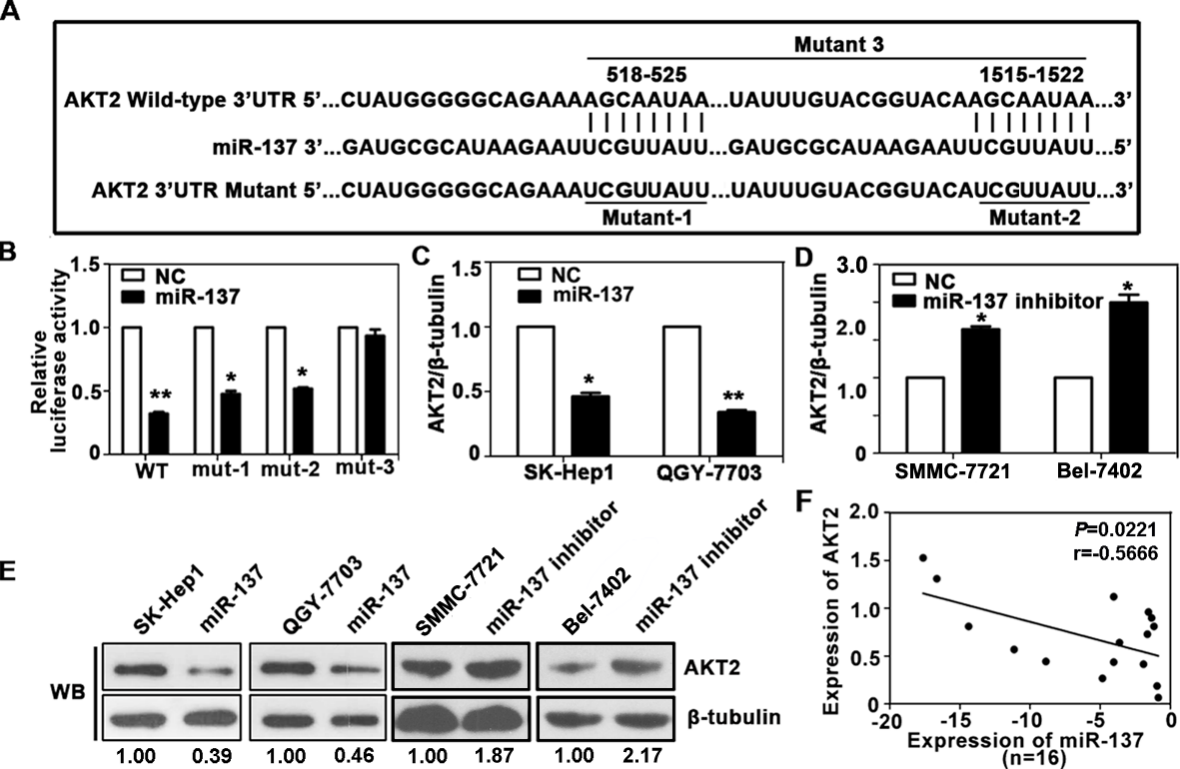
AKT2 is a direct target of miR-137 in HCC (A) Schematic of the construction of wild-type or mutant pGL3-AKT2 3'UTR vectors is indicated. (B) Relative luciferase activities were analysed in QGY-7703 cells. Renilla luciferase vector was used as an internal control. (C) Decrease in AKT2 mRNA expression by miR-137 was determined using qRT-PCR. (D) Increase in AKT2 mRNA expression by miR-137 inhibitor was determined using qRT-PCR. (E) Related expression of AKT2 protein in cells treated with miR-137 or its inhibitor was determined by western blot. (F) AKT2 protein expression was inversely correlated with miR-137 level in 16 pairs of HCC samples using linear regression models. The values of miR-137 were presented by the log_2_-fold change (HCC/NT). All *P<0.05, **P<0.01.

We next investigated the underlying mechanism of miR-137 deregulation in HCC cells. Among the 30 target interactions predicted using the search programs miRanda, miRtarget, PITA, RNAhybrid and Targetscan (Supplementary Table 3), AKT2 was selected for further validation due to its proliferation and metastasis properties. Two putative target sites were found, and the sequence of its binding site was highly conserved across different species (Fig. 3A and Supplementary Fig. S3A). To confirm the binding of miR-137 to the 3'-UTR of AKT2, we constructed vectors containing wild-type or mutant 3' UTR of AKT2 by directly fusing downstream of the firefly luciferase gene (Fig. 3A). A dual-luciferase reporter assay revealed that miR-137 significantly reduced the relative luciferase activity of wild-type AKT2 3'-UTR, whereas the luciferase activity of mutant AKT2 3'-UTR (mutant 3: containing both binding sites) remained unchanged (Fig. 3B). Furthermore, miR-137 blocked AKT2 expression at both the mRNA and protein level (Fig. 3C-E). The expression of AKT2 was increased in both HCC cell lines and clinical samples (Supplementary Fig. S3B-D). Interestingly, the basal levels of AKT2 in several HCC cell lines appeared to be reversely correlated with the endogenous levels of miR-137 (Supplementary Fig. S3B). An inverse correlation of miR-137 and AKT2 was observed in HCC tissues (Fig. 3F). Taken together, our data showed that miR-137 negatively modulated AKT2 expression by directly binding to its 3'UTR.

### miR-137 suppresses cell proliferation and migration via the suppression of AKT2

**Figure 4:**
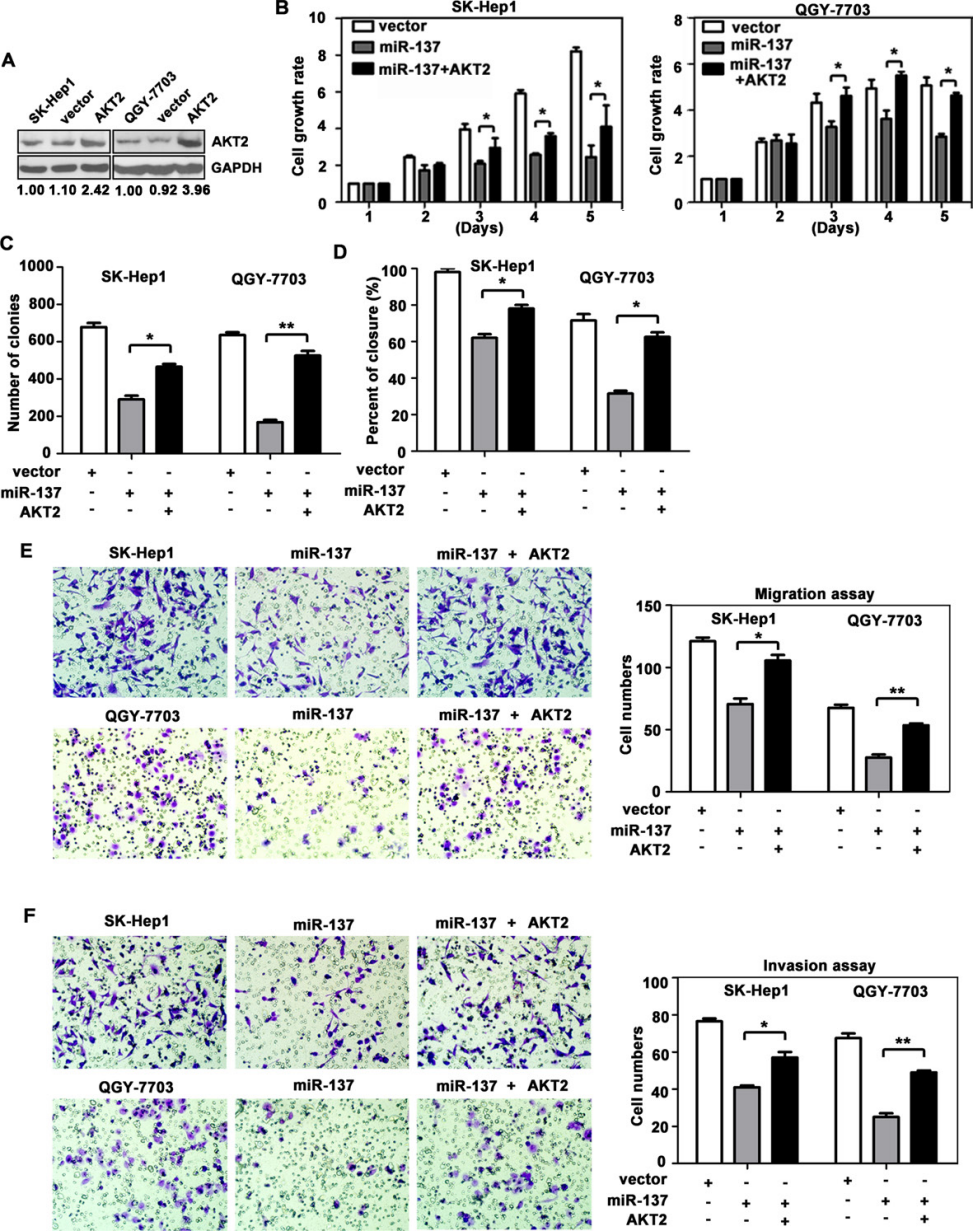
Reintroduction of AKT2 abrogates miR-137-induced suppression of HCC growth and metastasis (A) Re-expression AKT2 in HCC cells was examined using western blotting analyses. (B) AKT2 significantly repressed the miR-137-induced reduction of cell viability. Cells were transfected with miR-137 and AKT2 for specific time periods. The cell growth rates were determined using MTT assays. (C) A colony formation assay was performed to confirm the AKT2-mediated abrogation of miR-137-induced growth arrest in HCC. Cells expressing miR-137 and/or AKT2 were cultured for 14 days. Colonies were stained with 0.5% crystal violet and quantified. (D) Wound healing assays were performed on cells transfected with miR-137 and AKT2. The related percentages of wound closure are shown. (E, F) AKT2 reversed the inhibitory effects of miR-137 on cell migration and invasion. Cells transfected with miR-137 and/or AKT2 were transferred to transwell chambers in the absence (E) or presence (F) of Matrigel coating and incubated for 24 h. All *P < 0.05; ** *P* < 0.01.

We next confirmed whether AKT2 could affect the inhibitory effect of miR-137 on HCC progression. AKT2 was re-introduced in SK-Hep1 and QGY-7703 cells (Fig. 4A). MTT assays showed that reintroduction of AKT2 significantly abrogated the suppression of cell proliferation induced by miR-137 (Fig. 4B). miR-137-mediated loss of colony formation was also antagonised by forced expression of AKT2 (Fig. 4C). Furthermore, over-expression of AKT2 rescued the inhibition of cell migration and invasion induced by miR-137 (Fig. 4D-F). In addition, the alterations of proteins involved in EMT and AKT/mTOR signalling pathways were rescued by AKT2 over-expression (Supplementary Fig. S4).

**Figure 5:**
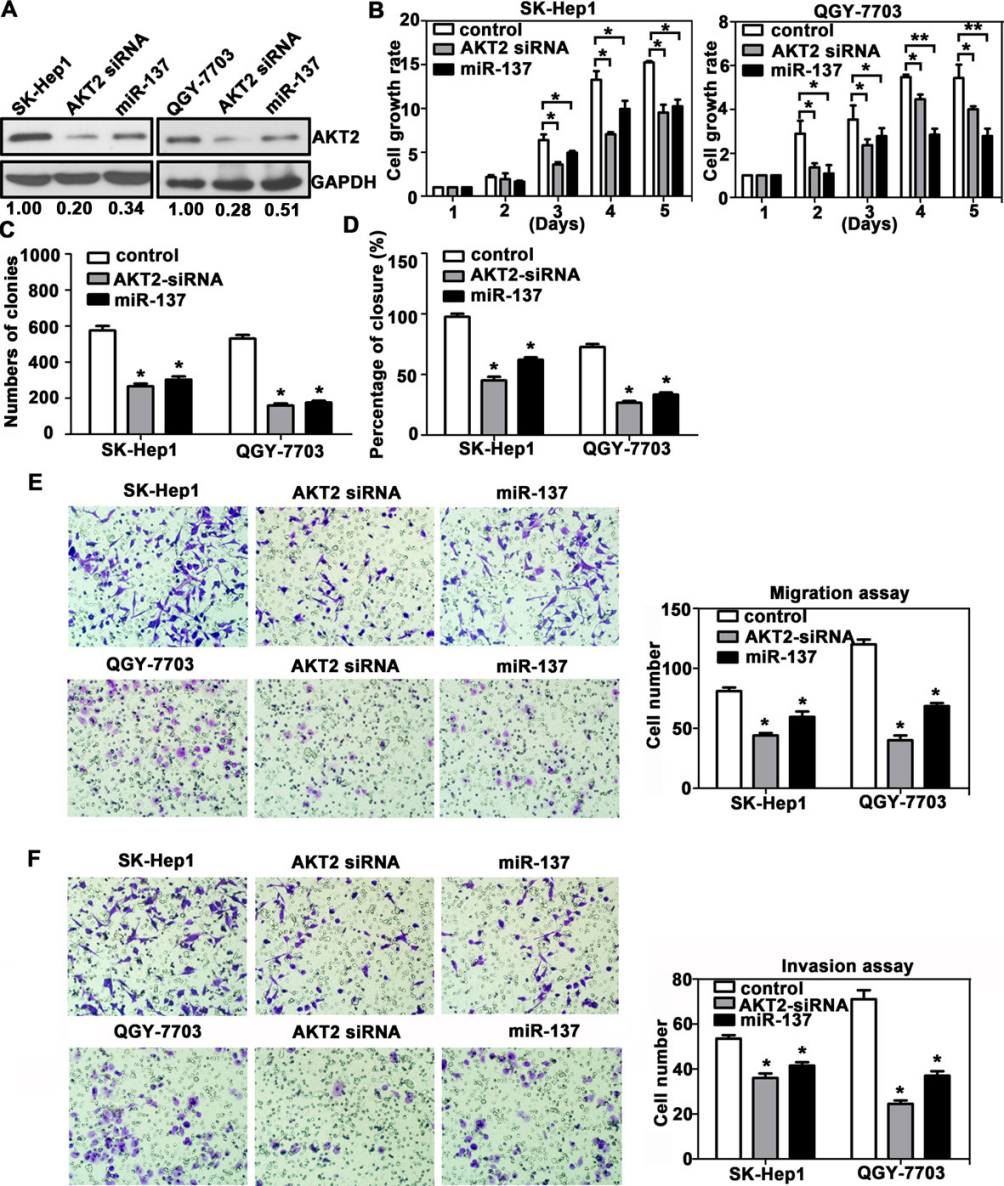
AKT2 silencing recapitulates the effects of miR-137 on HCC cells (A) AKT2 expression was silenced using AKT2 siRNA and miR-137 mimics. (B-F) The experiments described in Figure 4 were re-performed using AKT2 siRNA.

We also showed that silencing of AKT2 resulted in similar phenotype induced by miR-137 expression in HCC cells. AKT2 expression was decreased by miR-137 and AKT2 siRNA in SK-Hep1 and QGY-7703 cells (Fig. 5A). MTT and colony formation assays revealed that both miR-137 and AKT2 siRNA induced a comparable inhibition of cell growth (Fig. 5B&C). However, AKT2 siRNA could mimic the suppression of cell migration and invasion induced by miR-137 in both HCC cells (Fig. 5D-F). Collectively, our data indicated that miR-137 suppressed cell proliferation and migration by suppressing AKT2.

### miR-137 is directly regulated by the transcription factor FoxD3

**Figure 6:**
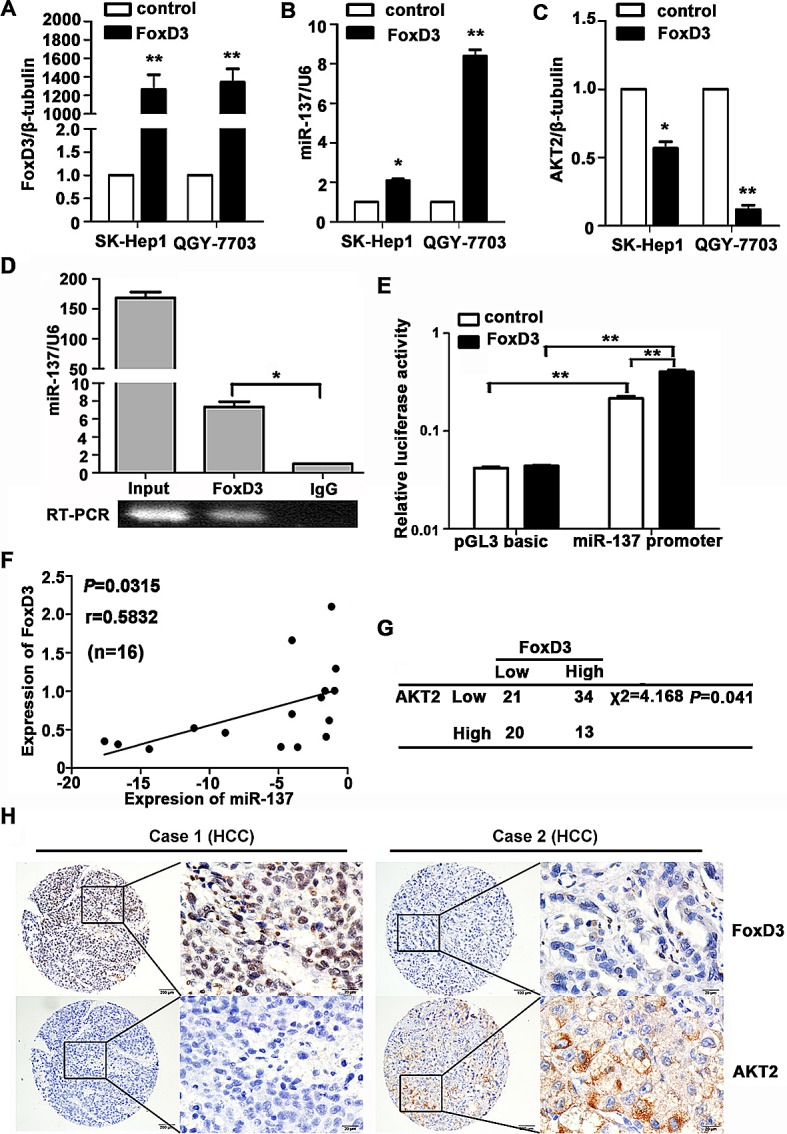
miR-137 is directly regulated by the transcription factor FoxD3 (A-C) Levels of FoxD3 mRNA, miR-137 and AKT2 mRNA were determined. HCC cells were transfected with FoxD3 and an empty vector for 24 h. The related expression of FoxD3 (A), miR-137 (B) and AKT2 (C) were examined using qRT-PCR. (D) FoxD3 directly bound to the promoter of miR-137. ChIP assay was performed in QGY-7703 cells transfected with a vector expressing FoxD3. qRT-PCR was performed using primers specific for −1485 to −1496 of the miR-137 promoter. (E) Luciferase activity of miR-137-luc construct after transfection of FoxD3 plasmid in QGY-7703. (F) FoxD3 protein expression was positively correlated with miR-137 in 16 pairs of HCC patients using linear regression models. The values of miR-137 were presented by the log_2_-fold change (HCC/NT). (G) FoxD3 expression was inversely correlated with AKT2 in HCC patients. The expression levels of FoxD3 and AKT2 in 88 HCC samples were determined using IHC. The median of IHC score was used as the cut-off value to divide patients into low and high expression groups. (H) The representative imagines were shown that IHC staining of FoxD3 and AKT2 in HCC. *P<0.05, **P<0.01.

To explore the regulation of miR-137 expression, we analysed the 1.5 kb region of miR-137 upstream. Many factors were predicted using 3 databases, and FoxD3 was selected for further validation due to its nature of a tumour suppressor (Supplementary Table 4-5). The expression of FoxD3 was first examined in HCC. Our results showed that FoxD3 expression was decreased in HCC cell lines and tissues (Supplementary Fig. S5A-C). FoxD3-induced increase of miR-137 was confirmed in HCC cells (Fig. 6A-B and Supplementary Fig. S5E). Decreases of AKT2 mRNA and protein levels were further determined (Fig. 6C and Supplementary Fig. S5D&E). CHIP assay was performed to demonstrate that FoxD3 could directly bind to the region of −1485 to −1496 in the miR-137 promoter (Fig. 6D). A dual-luciferase reporter assay showed that transient expression of FoxD3 effectively stimulated transcription of miR-137 in QGY-7703 cells (Fig. 6E). A positive correlation of FoxD3 and miR-137 was also revealed in HCC fresh tissues (Fig. 6F).TMA-based IHC staining showed a significant reverse association of FoxD3 and AKT2 expression in HCC patients (Fig. 6G-H). Taken together, these findings suggested transcription factor FoxD3 as a functional regulator of miR-137 in HCC.

### Ectopic expression of miR-137 inhibits tumour growth and metastasis *in vivo*

**Figure 7:**
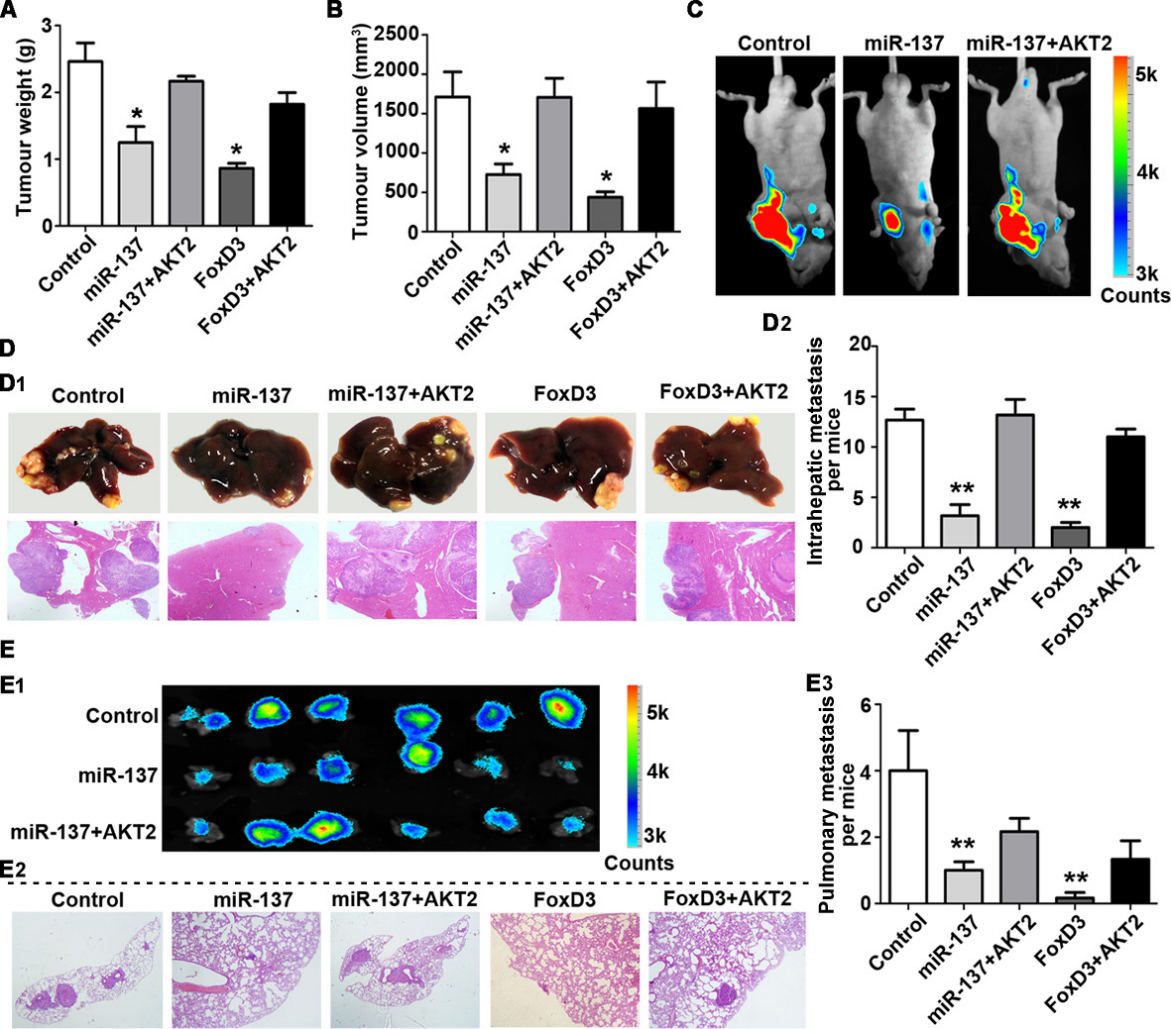
miR-137 inhibits HCC growth and metastasis via targeting AKT2 *in vivo* (A-C) miR-137 inhibits tumour growth via targeting AKT2 in HCC.1 X 10^7^ QGY-7703 cells of each group were injected into the right flank of nude mice. After 30 days, the tumours were weighed (A) and measured (B). The bioluminescence images of the liver tumours were obtained in the three indicated groups (C). (D, E) miR-137 inhibits tumour metastasis via targeting AKT2 in HCC. Representative images of the liver with metastatic nodules and H&E staining are shown for each group (D1). The average of the nodules in every liver was quantified (D2). Lung metastasis of HCC was identified using bioluminescence and H&E staining (E1-2). The mean of the metastatic nodules in the lung were measured (E3). All *P<0.05, **P<0.01.

We further confirmed the inhibitory effects of miR-137 on HCC *in vivo*. Compared with the control group, stable QGY-7703 and SK-Hep1 cells expressing miR-137 or FoxD3 generated tumours of lighter weight and smaller volume. Consistent with this *in vitro* data, re-expression of AKT2 markedly reversed the suppression of tumour growth induced by miR-137 (Fig. 7A-B, Supplementary Fig. S6A-B and Supplementary Fig. S7A-D). The hepatic weight ratio was shown in Supplementary Fig. S6C. The inhibitory effect of tumour growth by miR-137 restoration, which can be abrogated by AKT2 overexpression, was also demonstrated using bioluminescence (Fig. 7C).

The HCC metastatic model was used to confirm the effect of miR-137 on HCC metastasis. As shown by Figure 7D-E, re-expression of miR-137 and FoxD3 reduced the metastasis of HCC xenografts, which was remarkably rescued by AKT2. Compared to the control groups, liver metastatic nodules were significantly decreased in miR-137 and FoxD3 groups, but not in groups with AKT2 overexpression (Fig. 7D and Supplementary Fig. S7E-F). Furthermore, the lung metastasis was markedly suppressed by miR-137 and FoxD3, as shown using bioluminescence and histological analyses (Fig. 7E). The lung weight ratio was shown in Supplementary Fig. S6D. The expressions of AKT2, Ki67 (proliferative marker) and vimentin (mesenchymal marker) were consistent with our *in vitro* data (Supplementary Fig. S6E). Metastasis in the lymph node was also found in the control and AKT2 overexpression groups (Supplementary Fig. S8).

## DISCUSSION

Within the last decade, aberrant expression of microRNAs has been reported in human cancers [5] Several studies have shed light on tumour-targeting therapies using microRNA as a novel diagnostic and therapeutic tool [17]. In the present study, down-regulation of miR-137 was frequently observed in HCC tissues and may serve as an independent predictor for the outcome of HCC patients. Furthermore, ectopic miR-137 expression repressed HCC proliferation and migration *in vitro*, and tumour growth and metastasis *in vivo.* We further characterised AKT2 as a novel functional target of miR-137. Moreover, the transcription factor FoxD3 regulated the expression of miR-137, which in turn affected the progression of HCC. Our data suggest an important role of miR-137 in HCC progression.

The aberrant expression of miRNA has been evidence as a signature in hepatocarcinognesis. Toffanin et al. proposed a miRNA-based classification of three subtype of HCC: the wingless-style MMTV integration, interferon-related, and proliferation subclass [18]. Sato et al. developed a mathematical model to assess the risk of HCC recurrence after liver resection, according to miRNA expression profiling [19]. On the other hand, studies have also determined the prognostic value of miRNAs in HCC. Down-regulation of miR-122 was associated with poor prognosis in HCC [20]. Wong et al. reported that miR-139 decrease was correlated with poor outcome of metastatic tumours [21]. In this study, we found that miR-137 down-regulation was related with the invasive clinical features and unfavourable prognosis in HCC.

Proliferation and metastasis, two hallmarks of malignancy, are the leading causes for the cancer-related death [22]. Recent studies have shown that miRNAs are associated with these two events. miR-17-5p promoted HCC cell migration and proliferation by targeting P38-HSP27 pathway [7]. miR-26a inhibited HCC growth and metastasis via suppressing IL6 [6]. In the present study, we demonstrated that miR-137 exerted inhibitory effects on HCC progression by regulating AKT/mTOR pathway. Our results also showed that miR-137 re-expression resulted in decrease of p-AKT, p-mTOR, p-p70S6K, MMP2, and vimentin, but increase of E-cadherin, indicating that miR-137 was involved in HCC EMT process. Collectively, we demonstrated that miR-137 affected HCC progression via regulating EMT and AKT signalling pathways.

Our data suggest AKT2 as a direct downstream mediator of miR-137 in HCC. In human cancers, miR-137 can modulate the expression of a multitude of genes, including EZH2, FMNL2, and Paxillin [9, 23, 24]. Relationship between miR-137 expression and some well-known genes in HCC cells lines, such as paxillin and Met, have also determined in our study (Supplementary Fig. S9). In TargetScan database, many components involving the AKT pathway are putative targets of miR-137, such as PI3KR3, TGFA and Twist1. In our study, AKT2 was identified as a direct target of miR-137. AKT2 has been shown to be regulated by multiple miRNAs, e.g. miR-612, miR-203 and miR-184 [15, 25, 26]. miR-612, down-regulated in metastatic HCC, targeting AKT2 via 3' UTR regions distinct from those of miR-137, suppressed the invasive-metastatic cascades [25]. It should be of interest to study the potential synergistic inhibitory effects of combination of miR-612 and miR-137 on HCC progression.

miRNAs have been revealed to be regulated by transcription factors. Chang et al. showed miR-34a was directly transactivated by p53 to promote apoptosis in pancreatic cancer [27]. O'Donnell and colleagues reported that miR-17-5p and miR-20a were activated by c-MYC to negatively regulated E2F1 [28]. Liang et al. reported that miR-137 was regulated by HMGA1 [9]. In this study, we demonstrated that transcriptional factor FoxD3 modulated miR-137 expression. Positive correlation between FoxD3 and AKT2 was found in HCC cells and tissues. Interestingly, Liu et al. reported that FoxD3 was down-regulated in cells treated with AKT2 siRNA [29]. We also observed that miR-137 was down-regulated by P50 (NFκB1) in SK-Hep1 cells (data not shown).Ozes et al. showed that AKT was involved in the activation of NFκB [30]. The relationship of NFκB, FoxD3, miR-137 and AKT2 requires further investigation in future.

Collectively, our study provides experimental evidence that miR-137 suppresses cell growth and metastasis in HCC by directly targeting AKT2. The transcription factor FoxD3 induces the expression of miR-137, which subsequently inhibits the expression of AKT2. The newly identified FoxD3/miR-137/AKT2 signalling axis offers new insights into the pathogenesis of HCC and a potential therapeutic target for HCC treatment.

## MATERIALS AND METHODS

### Patients and Tissue Specimens

A total of 136 paraffin-embedded HCC specimens and 115 adjacent non-tumourous liver tissues were obtained from the Department of Pathology of Sun Yat-sen University Cancer Center from January 2004 to December 2011. Another group of 23 pairs of paraffin-embedded HCC tissues and corresponding HCC venous metastases was obtained from July 2012 to July 2013. An additional 16 matched fresh HCC tissues and adjacent non-tumourous liver tissues were collected from patients during hepatic resection. None of the patients had received radiotherapy or chemotherapy prior to the surgery. The use of human tissues was approved by the Sun Yat-sen University Cancer Center Institute Research Ethics Committee.

### Cell Lines and Cell Culture

PLC, Huh7, SK-Hep1 cancer cell lines were purchased from American Type Culture Collection (ATCC, Manassas, VA). L-02 (a immortalized liver cell line), SMMC7721, Bel-7402, QGY-7703, Bel-7404 HCC cell lines were obtained from the Cell Resource Center, Chinese Academy of Science Committee (Shanghai, China). These cell lines were maintained in RPMI-1640 medium supplemented with 10% heat-inactivated fetal bovine serum (FBS, Hyclone, Logan, UT). Cells were routinely maintained at 37°C in a humidified incubator containing 5% CO_2_.

### Quantitative Real-time PCR (qRT-PCR) and Western Blot Analysis

qRT-PCR was performed using miRCURY LNA™ universal cDNA synthesis Kit and miRCURY LNA™ SYBR Green master mix (Exiqon, Vedbaek, Denmark). Primers and antibodies used in western blot are described in the Supplementary Materials and Methods.

### Tissue Microarray (TMA) Construction and Immunohistochemistry

Details are described in the Supplementary Materials and Methods.

### Immunohistochemistry

The expression levels for AKT2 and FoxD3 in tumour tissues were determined by immunostaining with antibodies against AKT2 (1:50, SantzCruz, CA, USA) and FoxD3 (1:100, Millipore and Biolegend, Bedford, MA). The protein levels were determined by Semi-quantitative IHC detection. Using the H-score method, we multiplied the staining intensity score by the percentage score.

### Vector Construction and Transfection

A luciferase reporter assay was performed using the firefly luciferase-expressing vector psiCHECK2. To construct the psiCHECK2-AKT2-3'UTR-wt plasmid, a wild-type 3'UTR segment of AKT2 mRNA containing the putative miR-137 binding sites was amplified and cloned into the XhoI and NotI sites downstream of the luciferase reporter gene in psiCHECK-2. psiCHECK2-AKT2-3'UTR-mut carries the mutated sequence in the miR-137 binding sites. Lentivirus with the miR-137 expression vector (pLV3-miR-137) and control vector were (pLV3-control) purchased from GenePharma (Shanghai, China). Briefly, oligonucleotides encoding shRNA with mature miR-137 sequences (Supplementary Table 6) were subcloned into the BamHI and EcoRI sites of a lentiviral expression vector PGLV3/H1/GFP+ puro (GenePharm Co., Ltd.) and verified using DNA sequencing. Virus particles were harvested 48 h after cotransfection of the pLV3-miR-137 or pLV3-control with lentivirus packing vector into HEK-293T cells. The hsa-miR-137 mimic and control mimic (miR10000843), hsa-miR-137 inhibitor and control inhibitor (miR20000429) were purchased from Ribobio (Guangzhou, China). miR-137 inhibitor is a chemically modified, single stranded nucleic acids designed to specifically bind to and inhibits endogenous miR-137 molecules. miR-137 mimics/inhibitor was transfected into HCC cells at a final concentration of 100nM. SiRNAs were purchased from GenePharma (Shanghai, China) and the sequences of these siRNAs were provided in the Supplementary Table 6. Transfection was performed according to the manufacturer's protocol. The expression vector pcDNA3.1 containing FoxD3 was obtained from Prof. Francis K. L. Chan (Institute of Digestive Disease, Hong Kong). The AKT2 expression vector, and expression vector pcDNA 3.0 containing AKT2 was obtained from Prof. Joe Testa (Fox Chase Cancer Center, Philadelphia), which was used for “rescue” experiments.

### Luciferase Reporter Assay

For the binding of miR-137 to AKT2 3'UTR, the 3'UTR of AKT2 gene was amplified by PCR and inserted into the psiCHECK2 vector. Mutant constructs in miR-137 binding sites of AKT2 3'UTR region were generated. QGY-7703 cells were cotransfected with either miR-137 mimics or the negative control and psiCHECK2-AKT2-3'UTR-wt or psiCHECK2-AKT2-3'UTR-mut1-3. For the binding of FoxD3 to miR-137 promoter, the 1.5kb region directly upstream of miR-137 transcription binding site were amplified by PCR and inserted into the pGL3 vectors. QGY-7703 cells were cotransfected with either pcDNA3.1 or pcDNA3.1-FoxD3 and miR-137 promoter. After 48 hours of transfection, luciferase activity was analyzed according to the Dual-Luciferase Reporter Assay System (Promega, CA, USA), using a GloMax fluorescence reader (Promega, CA, USA).

### MTT and colony formation assays

Details are described in the Supplementary Materials and Methods.

### Wound Healing, Migration and Invasion assay

Details are described in the Supplementary Materials and Methods.

### Chromatin Immunoprecipitation Assay (CHIP)

The CHIP assay was performed using the CHIP Assay Kit (Thermo fisher scientific, USA). QGY-7703 cells infected with FoxD3 were lysed using SDS lysis buffer and DNA were sheared by sonication. Protein-DNA complexes were precipitated by control immunoglobulin G and anti-FoxD3 antibody respectively, followed by eluting the complex from the antibody. PCR was carried out with primers specific for miR-137.

### Animal Models

The HCC models in nude mice were constructed as described previously [31]. 1×10^7^ QGY-7703 cells stably infected with LV3-NC, LV3-miR-137, LV3-miR-137 plus pcDNA3.0-AKT2, pcDNA3.1-FoxD3, pcDNA3.1-FoxD3 plus pcDNA3.0-AKT2 vector, respectively named QGY-7703-control, QGY-7703-miR-137, QGY-7703-miR-137+AKT2, QGY-7703-FoxD3, QGY-7703-FoxD3+AKT2, were implanted into the flanks of male BALB/c-nude mice (n=6 per group, 3-4 weeks of age) subcutaneously. Bioluminescence signals were detected and imaged via a whole-body GFP imaging system from Zhongke Kaisheng Medical Technology Company (Guangzhou, China). Tumours were measured with callipers to estimate the volume from day 5 to day 30 after injection. Tumour sizes were evaluated using the formula: Volume (mm^3^) = [width^2^ (mm^2^) × length (mm)]/2. For *in vivo* tumour metastasis, the nude mice were anaesthetised and underwent a laparotomy (n=6 per group, BALB/c-nu, male, 3-4 weeks of age). 5*10^6^ cells were embedded into the left hepatic lobe using a microsyringe. After injection, the liver was returned to the abdominal cavity and closed with surgical drapes. Six weeks later, the mice were sacrificed and lung metastasis was determined in the fluorescent areas using an imaging system. Hepatic and lung metastases were detected using H&E staining and quantified by quantification of the metastatic lesions in each section. All animal studies were performed in the animal institute of Sun Yat-sen University Cancer Center according to the protocols approved by the Medical Experimental Animal Care Commission of Sun Yat-sen University Cancer Center.

### Statistical analysis

Statistical analysis was performed using the SPSS (version 16.0, Chicago, IL). Details are described in the Supplementary Materials and Methods.

## SUPPLEMENTARY MATERIALS AND METHODS TABLES AND FIGURES


