# Supplementary material for: FoxD3-regulated microRNA-137 suppresses tumour growth and metastasis in human hepatocellular carcinoma by targeting AKT2
Source: Oncotarget. 2014 Jun 10;5(13):5113–24. doi: 10.18632/oncotarget.2089 (PMC4148126; doi:10.18632/oncotarget.2089)
Supplement: Supplementary file 1 [file oncotarget-05-5113-s001.pdf]

FoxD3-regulated microRNA-137 suppresses tumour growth and metastasis in human hepatocellular carcinoma by targeting AKT2

Supplementary Materials and Methods

Quantitative Real-time PCR (qRT-PCR). qRT-PCR was performed using miRCURY LNA™ universal cDNA synthesis Kit and miRCURY LNA ™ SYBR Green master mix (Exiqon, Vedbaek, Denmark). Total RNA was extracted using the Trizol Reagent (Invitrogen, CA, USA). For miR-137 detection, reverse-transcribed cDNA was synthesised with the miRCURY LNA™ universal cDNA synthesis Kit (Exiqon, Vedbaek, Denmark). Quantitative RT-PCR (qRT-PCR) was performed with the miRCURY LNA ™ SYBR Green master mix (Exiqon, Vedbaek, Denmark) with the Stratagene Mx3000P Real-Time PCR system (Agilent Technologies, Inc., Santa Clara, CA, USA). Expression levels were normalised against the endogenous snRNA U6 control. The relative expression ratio of miR-137 in HCC specimens was calculated by the $2^{-\Delta\Delta CT}$ method. For mRNA analyses, cDNA was synthesised using Moloney murine leukaemia virus reverse transcriptase (Promega, Madison, WI, USA). RT-PCR was carried out with the following cycling conditions: 95°C for 10 min, 40 cycles of 94°C for 30 s, 60 °C for 30 s, 72°C for 30 s and a final extension of 10 min at 72°C. The sequences of the PCR primers are shown in Supplementary Table 6.

Western blot analysis. Total proteins were extracted and separated by 10% SEMS-PAGE and then transferred onto PVDF membrane (Millipore, Bedford, MA). The blotted membranes were incubated with anti-AKT2 (1:1000, Santa Cruz, CA, USA), anti-FoxD3 (1:1000, Millipore and Biolegend, Bedford, MA), anti-mTOR, anti-p70S6K, anti-4EBP1, anti-phospho-AKT2 (S474), anti-phospho-mTOR (S2448), anti-phospho-p70S6K (Thr389), anti-phospho-4EBP1 (Thr37/46) (1:1000, Cell Signaling Technology, CA, USA), anti-E-Cadherin, anti-N-Cadherin, anti-Vimentin, anti-MMP-2 (1:1000, Epitomics, CA, USA) and then probed with a secondary antibody (1:5000, Sigma, Cambridge, England). Anti-GAPDH (1:1000, Santa Cruz, CA, USA) was used as a loading control.

Tissue microarray (TMA) construction. In brief, all specimens were fixed in 4% formalin and embedded in paraffin. Two pathologists reviewed the corresponding HE-stained sections and mark out representative areas. Each tissue core with a diameter of 0.6 mm was punched from the marked areas and re-embedded, conducting in a tissue arraying instrument (Beecher Instruments, Sliver Spring, MD).

MTT and colony formation assays. After transfection, cells were seeded in 96-well plates (3×10^4 cells/ml) with 100 ul medium in each well and cultured for 5 days. MTT assay was performed by adding 20 ul of MTT (5mg/ml, AMRESCO, Solon, OH, USA) for 4 h at 37°C. Then, the formazan crystals were dissolved in DMSO (150 µl/well). The absorbance at 490 nm of each sample was measured using a multilabel plate reader (PerkinElmer). For the colony formation assay, 1000 cells were placed in 6-well plates and incubated at 37°C for 14 days. Clonies were fixed with methanol, stained with 0.1% crystal violet and counted.

Wound Healing, Migration and Invasion assay. Cells were seeded in six-well plates, after the cells reached 100% confluence. Wound healing assays were scratched with a sterile plastic tip. Medium was changed with serum-free medium and the cells were cultured for another 48 h. The percentage of wound closure was calculated for 3 randomly chosen fields. For the migration assay, 4×10^4 cells in serum-free medium were seed in the upper compartment of a transwell chamber (Corning; Lowell, MA). For the invasion assay, the chambers were previously coated with extracellular matrix gel (BD Biosciences, Sparks, MD). After incubation for 24-48 hours, the migrated or invaded cells on the lower membrane were counted after staining with 0.1% crystal violet and 20% methanol.

Statistical analysis. Using the Student's t-test, we analyzed the data for miR-137 expression in HCC specimens. Spearman correlation test was chosen for examining the correlations between miR-137 expression level and the clinical and pathological

variables. Survival curves were carried out by the Kaplan-Meier method and evaluated using the log-rank test. Identified factors were associated with survival by the cox proportional hazard regression model. Differences were considered significant for *P*-values less than 0.05.

Supplementary Table 1: Correlations of miR-137 and clinicopathological features of HCC patients.

Variable	miR-137 expression				
	**All cases**	**Low**	**High**	**χ2**	**P value[α]**
**Age (years) [b]**				0.03	0.863
< 48	63	32	31		
≥ 48	73	36	37		
**Gender**				0.605	0.437
Female	17	10	7		
Male	119	58	61		
**HBV infection**				0.085	0.771
Presence	123	61	62		
Absence	13	7	6		
**AFP (ng/ml)**				1.096	0.295
< 20	29	12	17		
≥ 20	107	56	51		
**Cirrhosis**				0.962	0.327
Presence	101	48	53		
Absence	35	20	15		
**Tumor size (cm)**				0.157	0.692
≤ 5	34	18	16		
> 5	102	50	52		
**Tumor nodule number**				2.584	0.108
Solitary	87	48	39		
Multiple (≥2)	49	20	29		
**Grade**				0.127	0.722
I-II	86	42	44		
III-IV	50	26	24		
**TNM stage**				0.030	0.862
I-II	79	39	40		
III-IV	57	29	28		
**Vein invasion**				5.303	**0.021**
Presence	29	9	20		
Absence	107	59	48		
**Involucrum**				4.329	**0.037**
Incomplete	78	33	45		
Complete	58	35	23		
**Distant metastasis**				7.908	**0.005**
Presence	22	17	5		
Absence	114	51	63		

[α]Chi-square test; [b] patients were divided according to the median age; AFP, alpha-fetoprotein.

Supplementary Table 2: Univariate and multivariate analysis of prognostic factors in 136 HCC patients.

Variable	Univariate analysis		Multivariate analysis	
	HR (95% CI)	P value	HR (95% CI)	P value
Age (years)	1.115(0.668-1.862)	0.988		
Gender	0.977(0.709-1.403)	0.677		
HBV infection	0.946(0.531-1.685)	0.849		
AFP (ng/ml)	1.486(0.982-2.249)	0.061		
Cirrhosis	0.953(0.646-1.407)	0.810		
Tumor size (cm)	1.320(0.892-1.952)	0.165		
Tumor multiplicity	1.569(1.100-2.238)	**0.013**	1.252(0.858-1.825)	0.243
Grade	1.195(0.888-1.609)	0.239		
TNM stage	1.329(1.093-1.616)	**0.004**	1.234(1.002-1.521)	**0.048**
Vein invasion	1.783(1.177-2.701)	**0.006**	1.544(0.991-2.407)	0.055
Capsule formation	0.784(0.566-1.106)	0.166		
metastasis	1.052(0.660-1.676)	0.831		
Recurrence	0.890(0.632-1.255)	0.507		
miR-137	0.627(0.440-0.893)	**0.010**	0.624(0.437-0.892)	**0.010**

AFP, alpha-fetoprotein; CI, confidence interval; HR, hazard ratio.

Supplementary Table 3: miR-137 target interactions predicted by miRanda, miRtarget, pita, RNAhybrid, and targetscan.

Refseq	Symbol	Description
NM_022373	HERPUD2	HERPUD family member 2
NM_016018	PHF20L1	PHD finger protein 20-like 1
NM_020774	MIB1	mindbomb homolog 1 (Drosophila)
NM_182526	C14orf83	chromosome 14 open reading frame 83
NM_032468	ASPH	aspartate beta-hydroxylase
NM_152446	C14orf145	chromosome 14 open reading frame 145
NM_001626	AKT2	v-akt murine thymoma viral oncogene homolog 2
NM_001004065	AKAP2	A kinase (PRKA) anchor protein 2
NM_001014435	CA7	carbonic anhydrase VII
NM_001017980	LOC203547	hypothetical protein LOC203547
NM_001415	EIF2S3	eukaryotic translation initiation factor 2, subunit 3 gamma
NM_001080779	MYO1C	myosin IC
NM_001080744	DGKG	diacylglycerol kinase, gamma
NM_001076786	QSER1	glutamine and serine rich 1
NM_001042546	ATPAF1	ATP synthase mitochondrial F1 complex assembly factor 1
NM_001042406	HMGCLL1	3-hydroxymethyl-3-methylglutaryl-Coenzyme A lyase-like 1
NM_001031812	CSNK1G3	casein kinase 1, gamma 3
NM_001024948	FNBP1L	formin binding protein 1-like
NM_000267	NF1	neurofibromin 1
NM_015272	RPGRIP1L	RPGRIP1-like
NM_014616	ATP11B	ATPase, class VI, type 11B
NM_014988	LIMCH1	LIM and calponin homology domains 1
NM_014663	JMJD2A	jumonji domain containing 2A
NM_006160	NEUROD2	neurogenic differentiation 2
NM_005955	MTF1	metal-regulatory transcription factor 1
NM_006999	POLS	polymerase (DNA directed) sigma
NM_006920	SCN1A	sodium channel, voltage-gated, type I, alpha subunit
NM_006624	ZMYND11	zinc finger, MYND domain containing 11
NM_006621	AHCYL1	S-adenosylhomocysteine hydrolase-like 1
NM_006618	JARID1B	jumonji, AT rich interactive domain 1B

Supplementary Table 4: The candidate transcription factors of miR-137 by different transcription factor prediction database.

Database	Transcription factor	Sequence	From	To	Score	Strand
Consite	c-REL	CGGTATTTCC	7	16	10.653	+
Consite	HFH-2/FoxD3	GATTTTTTTTT	348	359	12	+
Consite	Hunchback	TTTTTTTTA	351	360	10.028	-
Consite	TBP	CCGTTTCTTTTATAT	384	398	11.669	-
Consite	HMG-I/Y	GTTTTTGCTTTTCTTT	471	486	13.876	-
Consite	bZIP910	ACGTCAG	715	721	11.694	-
Consite	Tal1beta-E47S	GACAGATGGTGT	910	921	12.635	-
Mapper 2.0	DSF	-	217	227	9.3	+
Mapper 2.0	HMG-I/Y	-	471	485	6.4	-
Mapper 2.0	bZIP910	-	715	721	5.2	-
Tred	HFH-2/FoxD3	GATTTTTTTTTT	347	358	8.9	+
Tred	c-REL	-	-	-	8.06	+
Tred	Tal1beta-E47S	TCGACATATGTT	570	581	7.38	-
Tred	Hunchback	GGAAAAAAAAAT	330	339	6.84	-
Tred	HMG-I/Y		-	-	4.84	-
Tred	TBP	TTTTATATGGCAGAG	390	404	4.81	-
Tred	bZIP910	-	-	-	0	-

Supplementary Table 5: Location and sequence of FoxD3 binding sites.

Transcription factor	Sequence	From	To	Score	Strand
HFH-2/FoxD3	ATACAAATATAT	488	499	9.191	-
HFH-2/FoxD3	AAATAAACACTC	614	625	12.124	-
HFH-2/FoxD3	CAATGTTTAATT	840	851	11.265	+
HFH-2/FoxD3	AAATATTATTTT	946	957	11.002	+
HFH-2/FoxD3	TCTTATTTTTTT	1406	1417	9.574	+
HFH-2/FoxD3	AAATCAACATGA	1485	1496	9.743	-
HFH-2/FoxD3	AAACCAATATAG	1559	1570	10.177	-
HFH-2/FoxD3	AATAAAACAAAT	1643	1654	11.459	-
HFH-2/FoxD3	AAACAAATAAAT	1647	1658	13.017	-
HFH-2/FoxD3	AAATAAATAAGC	1651	1662	11.439	-
HFH-2/FoxD3	AAAAAAAAAATT	1722	1733	10.876	
HFH-2/FoxD3	AAAAAAAAATTC	1723	1734	12.117	
HFH-2/FoxD3	GTACATTTGCTT	1819	1830	9.287	

Supplementary Table 6: The sequences for primers and siRNAs used in the study.

Identifier Type	Sense sequence(5'-3')	Antisense sequence(5'-3')
LV3-NC	TTCTCCGAACGTGTCACGTTTC	
Hsa-miR-137	TTATTGCTTAAGAATACGCGTAG	
FoxD3	GCCCAAGAACAGCCTAGTGA	GTGAGAGGTTGTGGCGGATG
AKT2	CCCGGTTTTATGGTGCAGAGAT	GGCCGCACATCATCTCGTACAT
β-actin	CACCATGAAGATCAAGATCATTGC	GGCCGGACTCATCGTACTCCTGC
PXN	ACGTCTACAGCTTCCCCAACAA	AGCAGGCGGTCGAGTTCA
c-Met	CATGCCGACAAGTGCAGTA	TCTTGCCATCATTGTCCAAC
NC siRNA	UUCUUCGAACGUGUCACG	ACGUGACACGUUCGGAGAATT
AKT2-siRNA	GGGCUAAAGUGACCAUGAATT	UUCAUGGUCACUUUAGCCCTT
FoxD3-siRNA	AGACGGCGCUCAUGAUGCATT	UGCAUCAUGAGCGCCGUCUTT
Chip-miR-137	TAATACTTCTCCGGGCACAC	ACAGTCTGGTAGTTGGGCTA

Abbreviations: NC, negative control;

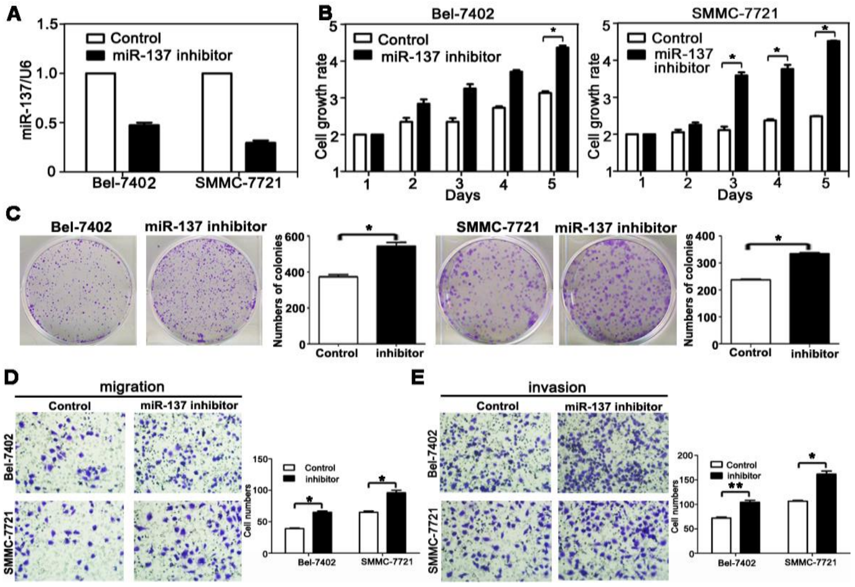

Figure S1: miR-137 inhibitor promotes HCC cells growth, migration and invasion *in vitro*. (A) Fold changes of mature miR-137 expression was determined in Bel-7402 and SMMC-7721 cells transfected with miR-137 inhibitor and negative control. **(B)** The cell growth rates were determined by MTT assays. **(C)** Clonies formed by HCC cells treated with miR-137 inhibitor or negative control were calculated and depicted. The values indicate the Mean+SEM of three independent experiments. **(D, E)** Migration and invasion assays of HCC cells with miR-137 inhibitor or negative control. Representative images were shown, and the quantification of three randomly selected fields was indicated. Data are the mean ± SEM. *P<0.05, **P<0.01.

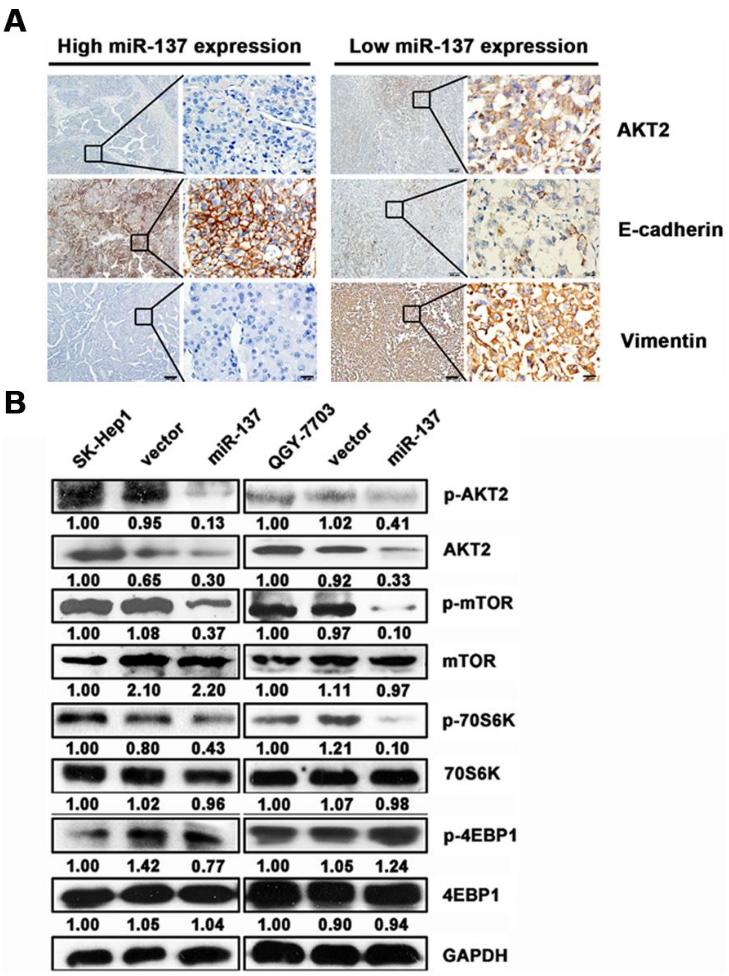

Figure S2: miR-137 is involved in EMT process and AKT2/mTOR pathways in HCC. (A) The levels of AKT2, E-cadherin and vimentin in HCC patients with low or high miR-137 expression were determined using IHC. **(B)** The expression of AKT2, mTOR, p70S6K, 4EBP1 and their phosphorylated forms in HCC cells with miR-137 overexpression were detected.

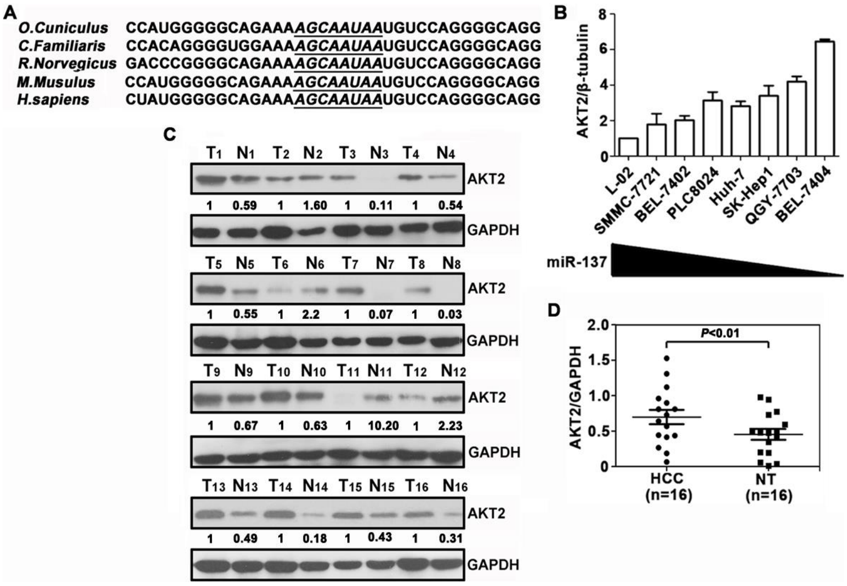

Figure S3: AKT2 is direct target of miR-137 in HCC. (A) The potential binding sequences for miR-137 within the AKT2 3'UTR of rabbit (*O. ouniculus*), dog *(C. familiaris*), rat (*R. norvegicus*), mouse (*M. musculus*) and human (*H. sapiens*). Seed sequences were leaned and underlined. **(B)** mRNA level of AKT2 in HCC cell lines were examined. The relationship of AKT2 mRNA and miR-137 was indicated. **(C)** AKT2 expression in the 16 paired HCC tissues and the corresponding nontumourous tissues were determined by western blot. The intensity for each band was densitometrically quantified. N, noncancerous tissue; T, HCC tissue. **(D)** AKT2 protein expression was higher in HCC compared to non-tumourous tissues. Data were shown according to the Intensity of AKT2 normalised against GAPDH.

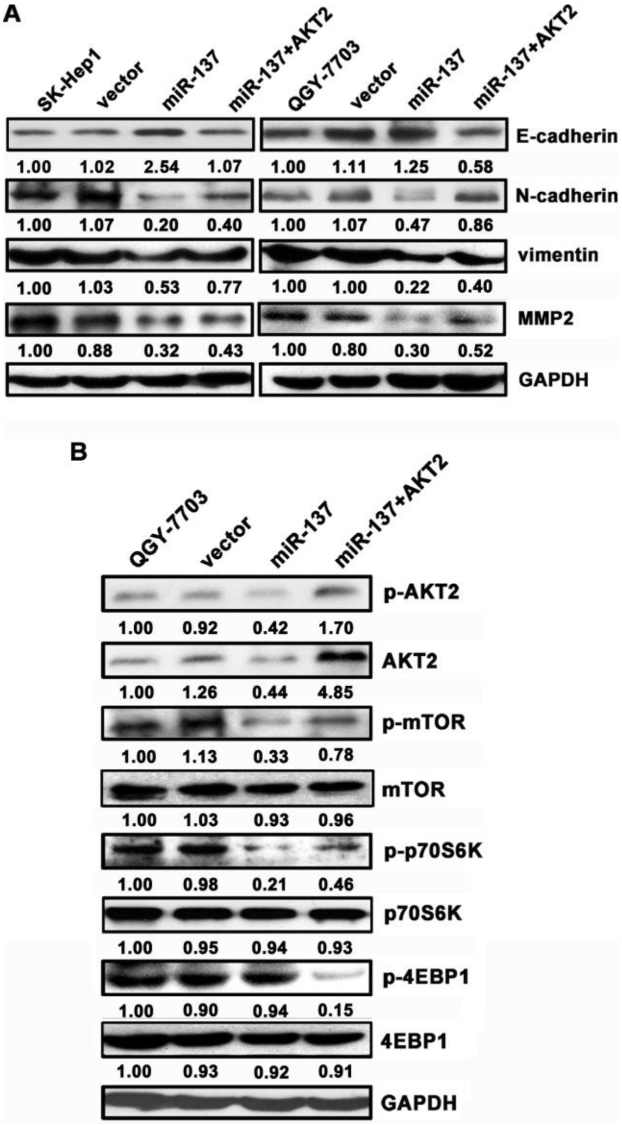

Figure S4: miR-137 is involved in EMT process and AKT2/mTOR pathways via AKT2 in HCC. (A) AKT2 overexpression rescued the miR-137-induced alterations of EMT markers. Expressions of E-cadherin, N-cadherin, vimentin, MMP2 in SK-Hep1 and QGY-7703 cells expressing miR-137 and/or AKT2 were examined by western blot. **(B)** Levels of AKT2, mTOR, p70S6K, 4EBP1 and their phosphorylated forms were determined in QGY-7703 cells transfected with miR-137 and/or AKT2.

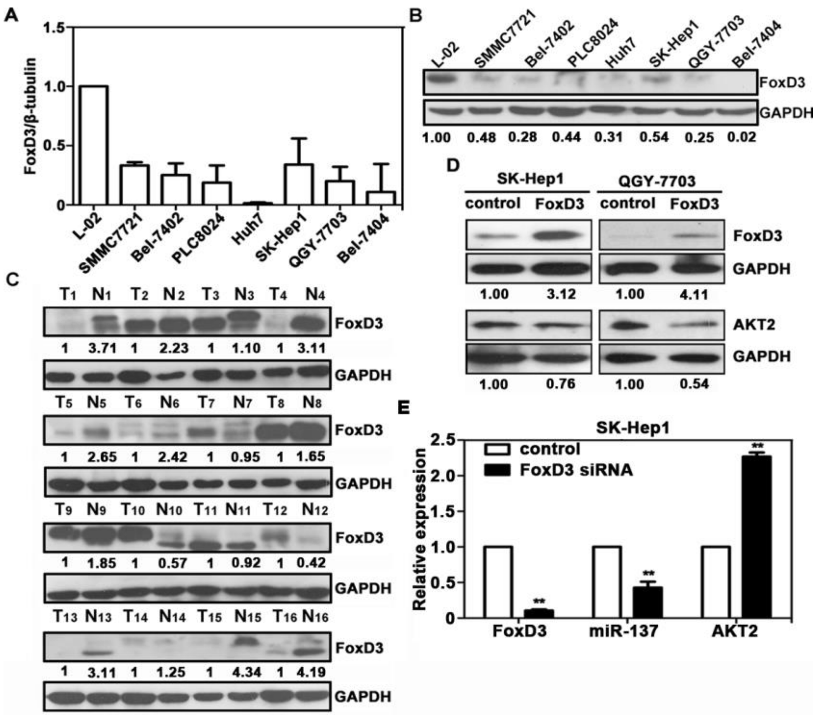

Figure S5: FoxD3 is down-regulated in HCC. (A, B) FoxD3 was decreased in HCC cell lines at both mRNA (A) and protein (B) levels, compared to the immortalized liver cell (L-02). **(C)** FoxD3 expressions in the 16 paired HCC tissues were determined. N, noncancerous tissue; T, HCC tissue. **(D)** HCC cells were transfected with FoxD3 and an empty vector for 24 h. The related protein levels of FoxD3, and AKT2 were examined by western blot. **(E)**The relative expressions of FoxD3, miR-137 and AKT2 in SK-Hep1 cells with FoxD3 knockdown were examined by qRT-PCR. ***P*<0.01.

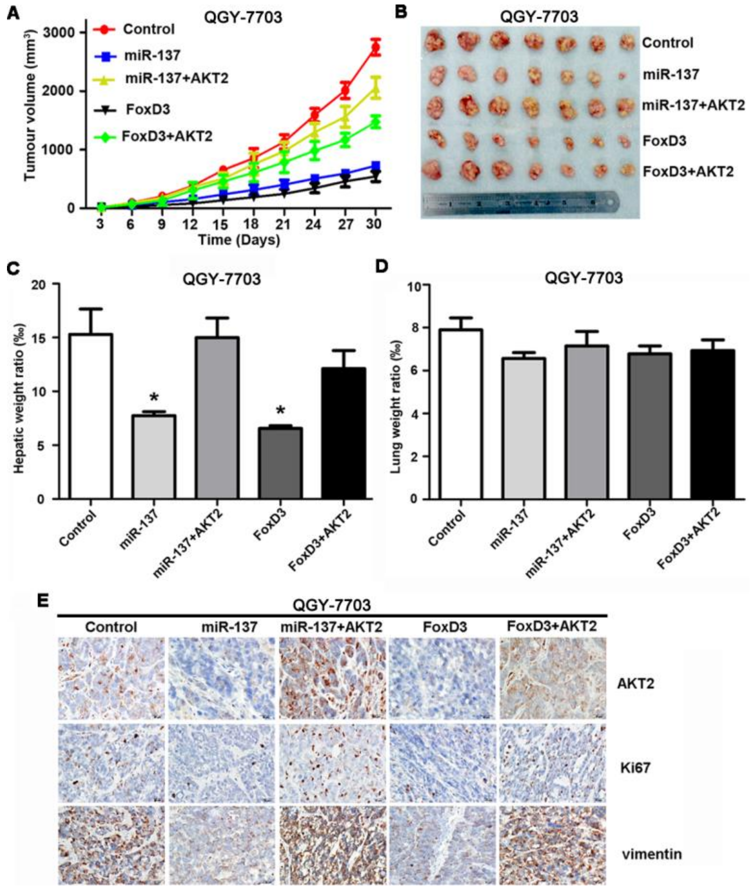

Figure S6: miR-137 inhibits QGY-7703-bearing tumour growth and metastasis *in vivo*. (A) The tumour volumes were recorded every three days. **(B)** After 30 days, the QGY-7703 bearing xenografts were resected and pictured. Liver **(C)** and lung **(D)** weight ration, comparing with the body, were measured at the sixth weeks after the orthotopic liver inoculation of nude mice. **(E)** The expression of Ki67 (proliferative marker) and vimentin (mesenchymal marker) was examined. *P<0.05.

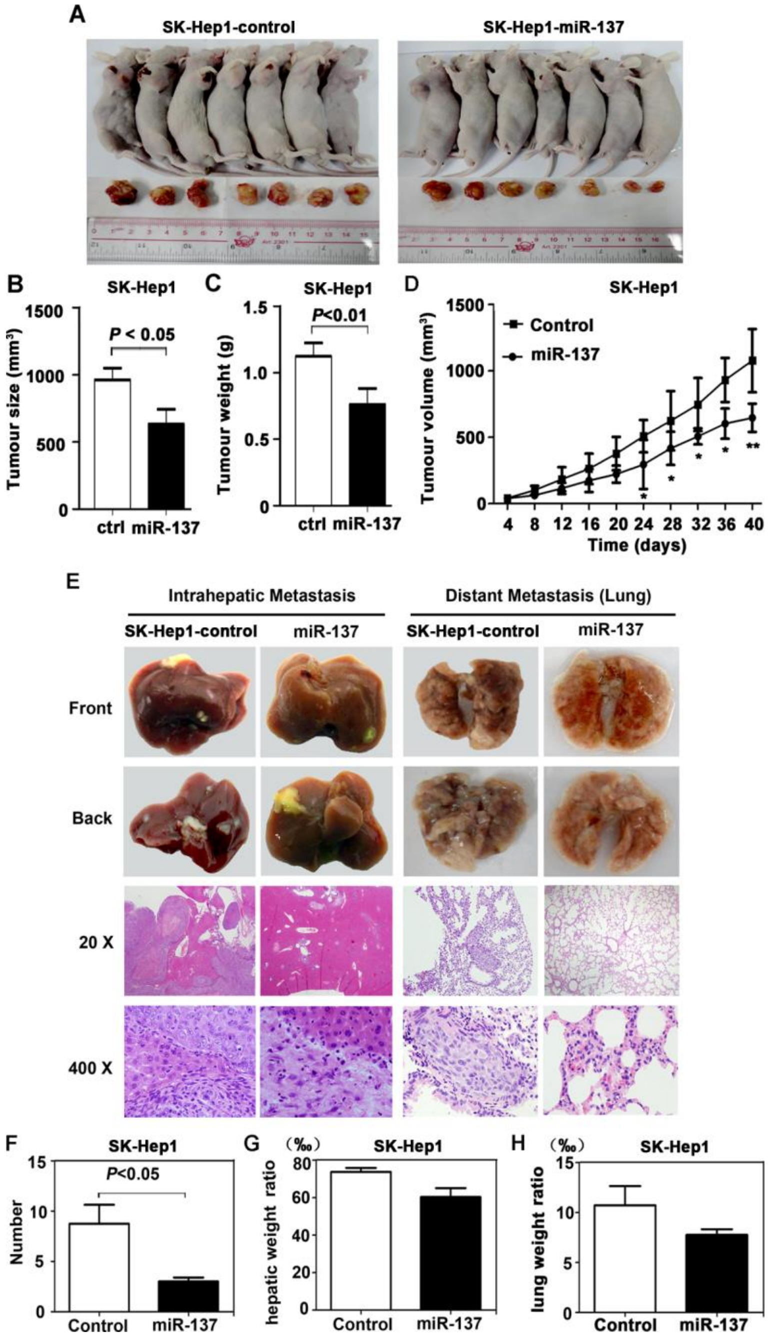

Figure S7: miR-137 inhibits SK-Hep1-bearing tumour growth and metastasis *in vivo*. Tumour images **(A)**, tumour size **(B)**, tumour weight **(C)** and tumour growth curves **(D)** of subcutaneous implantation models of HCC were shown. **(E)** Gross photo and HE stained sections of intrahepatic metastatic nodules and distal metastatic nodules in the lung at the sixth weeks after the orthotopic liver inoculation of nude mice were presented. **(F)** The number of metastatic nodules in the livers of each mice was counted. Liver **(G)** and lung **(H)** weight ration, comparing with the body, were measured. *P<0.05, ** P<0.01.

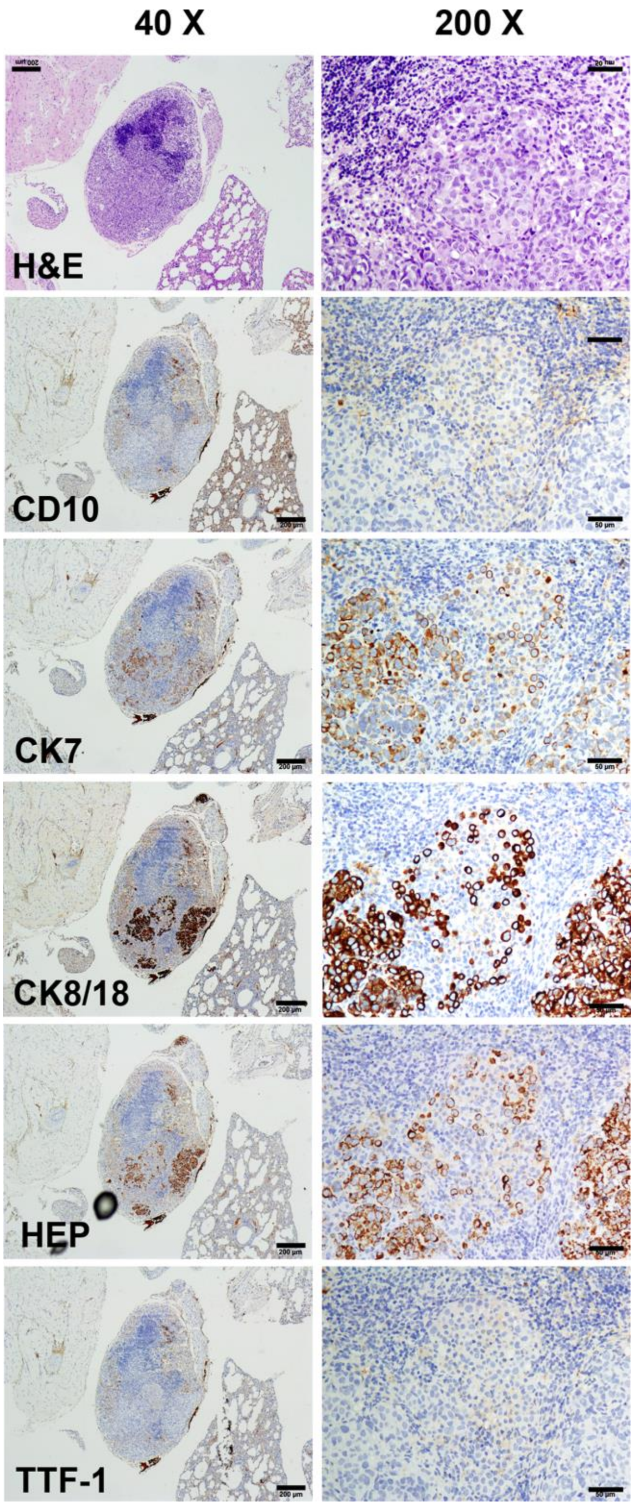

Figure S8: H&E and IHC stained sections of metastatic nodules in lymph node in QGY-7703-LV3-control group are shown.

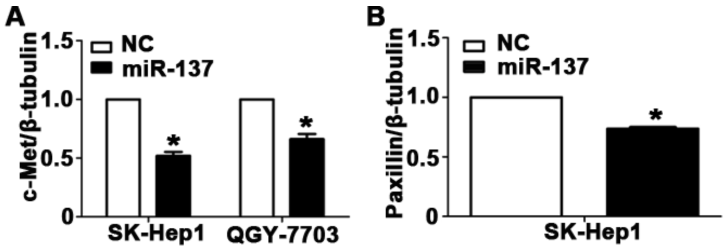

Figure S9: Paxillin and c-Met are direct targets of miR-137 in HCC. (**A-B**) Decrease in Paxillin and c-Met mRNA expression by miR-137 was determined using qRT-PCR. *P<0.05.
